# Weighted Gene Networks Derived from Multi-Omics Reveal Core Cancer Genes in Lung Cancer

**DOI:** 10.3390/biology14030223

**Published:** 2025-02-20

**Authors:** Qingcai He, Zhilong Mi, Ziqiao Yin, Zhiming Zheng, Binghui Guo

**Affiliations:** 1 School of Mathematical Sciences, Beihang University, Beijing 100191, China; 2 LMIB and SKLCCSE, Beihang University, Beijing 100191, China; 3 Shen Yuan Honors College, Beihang University, Beijing 100191, China; 4 Institute of Artificial Intelligence, Beijing Advanced Innovation Center for Future Blockchain and Privacy Computing, Beihang University, Beijing 100191, China; 5 Zhongguancun Laboratory, Beijing 100094, China

**Keywords:** weighted gene network, maximum entropy, markov chain entropy, DNA methylation, multi-omics

## Abstract

Lung cancer remains the leading cause of cancer-related deaths worldwide, driven by its complexity and the heterogeneity of its subtypes, which influence pathogenesis, tumor microenvironment, and genetic alterations. We developed a novel weighted gene regulatory network reconstruction method based on maximum entropy and Markov chain entropy principles, which integrates gene expression and DNA methylation data to generate biologically informed networks. Applied to LUAD and LUSC datasets, we define a network methylation index to determine whether gene methylation acts as oncogenic or tumor-suppressive. By revealing a stable core set of pathogenic genes, we identify not only genes with significant expression changes, such as *CD74* and *HGF*, but also pathogenic genes with stable expression, such as *BRAF* and *KDM6A*. Additionally, we uncover potential driver genes, such as *CORO2B* and *C20orf194*, associated with disease stage, gender, and smoking status. This method offers a more comprehensive understanding of NSCLC mechanisms, paving the way for improved therapeutic strategies.

## Simple Summary

Lung cancer remains a leading cause of cancer-related deaths worldwide, characterized by high heterogeneity and complex gene regulatory mechanisms. We developed a novel weighted gene regulatory network reconstruction method that integrates gene expression and DNA methylation data, leveraging principles of maximum entropy and Markov entropy. Applied to LUAD and LUSC datasets, our approach successfully identified a stable core set of pathogenic genes, including both highly expressed genes (e.g., *CD74*, *HGF*) and stably expressed genes (e.g., *BRAF*, *KDM6A*), which play critical roles in cancer progression. Furthermore, we uncovered methylation-driven oncogenes and tumor suppressors, revealing their dual roles in lung cancer development. By incorporating clinical variables such as disease stage, gender, and smoking status, we identified potential driver genes associated with specific patient subgroups, providing insights into personalized therapeutic strategies. This method not only enhances our understanding of lung cancer biology but also offers a robust framework for identifying novel therapeutic targets and advancing precision medicine.

## 1. Introduction

Lung cancer, as the leading cause of cancer deaths worldwide, accounted for 12.4% of new cases among 36 types of cancers, with over 1.8 million deaths, representing 18.7% of the global cancer mortality rate [1,2]. In-depth studies of lung cancer’s biological mechanisms have revealed its complexity, highlighting how the heterogeneity of different subtypes contributes to significant variations in pathogenesis, tumor microenvironment, and gene mutations [2,3,4,5], while its rising incidence among women and non-smokers underscores the influence of factors beyond smoking, including other internal and external contributors [6]. Building on this complexity, AI methods have shown promise in predicting the effects of DNA mutations, PPI, and disease [7,8], advancing precision medicine and initiatives like the Human Genome Project II [9]. However, as these methods rely on mathematical models that often lack interpretability, developing innovative, interpretable models is essential for understanding and addressing cancer mechanisms.

Barabasi’s concept of network biology emphasizes the systematic classification of cellular molecules and interactions in understanding their roles in complex systems [10]. The complex network approach focuses on identifying key genes and applying this understanding to cancer biology, disease progression prediction, and therapeutic target identification, such as DNB [11,12]. The most frequently employed methodologies include entropy metrics, topological analysis, cluster analysis, and so forth [13,14,15]. Research on gene regulatory networks associated with Müller glial cells across species has identified methods for inducing neuronal regeneration after injury in adult mice, offering potential strategies for treating retinal neuron loss caused by degenerative diseases [16]. Studies on growing cells have demonstrated that biological networks are more stable than random networks, suggesting that evolutionary stability constraints influence network structure [17]. Additionally, the use of Markov chain entropy to assess single-cell differentiation potential has enabled the identification of normal and cancer stem cell phenotypes [18]. However, while these approaches have provided valuable insights, they often focus on single-omics data, limiting their ability to capture the multifaceted nature of biological systems. Currently, methods such as WGCNA are widely used for constructing weighted co-expression networks, but they primarily rely on expression data, and there is comparatively little research on weighted gene regulatory networks [19].

To better understand the mechanisms of cancer driver genes, recent multi-omics integration studies have highlighted the importance of combining diverse molecular layers, including genomics, transcriptomics, and epigenomics [20,21]. While early and intermediate integration strategies address this by pre-integrating datasets, most machine learning models struggle to handle the large matrices generated by early integration, and intermediate integration often relies on unsupervised matrix factorization, which makes it difficult to incorporate extensive prior biological knowledge [22,23]. Concretely, among epigenetic modifications, DNA methylation plays a critical role in regulating gene expression and has emerged as a key factor in cancer development and progression [24]. However, many current network construction approaches overlook the impact of epigenetic factors, relying predominantly on single-omics data, such as gene expression [25]. This limitation can lead to models that fail to capture the dynamic interplay between gene expression and epigenetic regulation, particularly in complex diseases like lung cancer, where methylation-driven changes in gene expression are critical for understanding disease progression and identifying therapeutic targets. Integrating DNA methylation into network modeling, along with other omics data, is crucial for developing more accurate representations of cancer mechanisms, offering deeper insights into cancer driver genes and opening new avenues for research and therapeutic strategies [26].

To explore the dynamic interplay between gene expression and epigenetic regulation, we propose a novel weighted gene regulatory network reconstruction method that integrates gene expression and DNA methylation data, leveraging known gene regulatory relationships from the BioGRID database. By combining maximum entropy and Markov chain entropy principles, our approach not only captures the functional relevance of pathogenic genes but also quantifies the impact of methylation on gene regulation. We applied this method to LUAD and LUSC datasets, constructing an original network $G_{0}$ and a methylation-specific network $G_{1}$ for each sample. By defining a network methylation index, we analyzed the impact of DNA methylation on known pathogenic genes throughout disease progression, focusing on genes with missing network methylation indices in most samples. Furthermore, we calculated the weighted PageRank scores for each sample and clustered genes using the K-means algorithm to identify a stable core set of pathogenic genes unaffected by methylation. By incorporating disease stages, gender, and smoking status, we identified potential pathogenic genes, offering novel insights into their roles in lung cancer pathogenesis.

## 2. Materials and Methods

**Figure 1:**
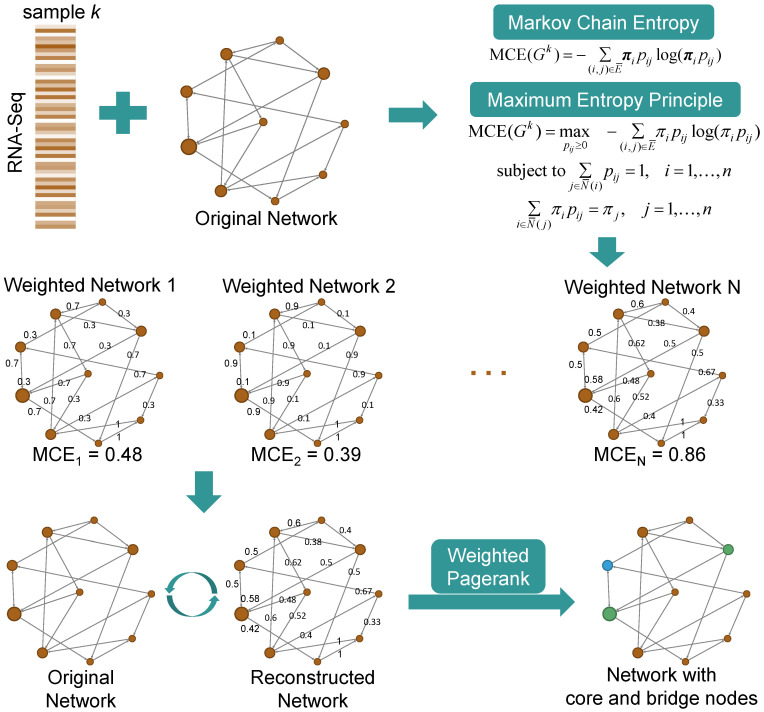
Schematic diagram of the flow of the research methodology. This figure illustrates the maximum entropy network reconstruction and clustering to find the core gene module, and the flow of arrows shows the incremental analysis flow from raw data preprocessing to core pathogenic gene mining.

This study proposes a novel methodology for reconstructing weighted gene regulatory networks by integrating gene expression and DNA methylation data, as shown in Figure 1. The approach is based on the principles of maximum entropy and Markov chain entropy (Section 2.2), which are used to model probabilistic transitions between genes while maximizing the network’s entropy under biological constraints (Section 2.3). The method constructs two distinct networks—an original network $G_{0}$ (unmethylated) and a methylation-specific network $G_{1}$—enabling the quantification of DNA methylation’s impact through a network methylation index. Key steps include the calculation of Markov Flow Entropy (Section 2.4) to assess methylation-driven gene interactions, the application of a weighted PageRank algorithm (Section 2.5) to identify critical genes, and clustering analysis to uncover stable core pathogenic genes. This integrated framework provides a comprehensive understanding of lung cancer mechanisms and identifies potential therapeutic targets.

### 2.1. Dataset

The gene regulatory network comes from the BioGrid database [27]. We used the gene expression data and methylation beta value data of LUAD and LUSC in the TCGA project, and then screened samples with both data for analysis. Specific sample information is shown in the following table. We excluded samples with missing clinical information (e.g., smoking status or disease stage) from the analyses that required these variables. This ensures that the results are based on complete and reliable data, see Table 1.

### 2.2. Markov Chain Entropy

A Markov chain is a stochastic model that describes a system transitioning between states, where each transition depends only on the current state, not the history [28]. This property, known as the Markov property, makes it ideal for modeling dynamic processes. In gene regulatory networks, Markov chains can capture the probabilistic transitions of gene states, such as activation or repression, based on regulatory interactions, providing a framework for analyzing and simulating complex biological dynamics. A Markov chain satisfies the following conditions, when given the following probability vector:

(1) $$v=\left(v_{0},v_{1},\cdots ,v_{t-1}\right),\;v_{i}>0,\;\sum_{i=0}^{t-1}v_{i}=1$$

and matrix

(2) $$\left.P=\left[\begin{array}{ccc} p_{00} & \cdots & p_{0,t-1}\\ \vdots & \; & \vdots \\ p_{t-1,0} & \cdots & p_{t-1,t-1} \end{array}\right.\right],\;p_{ij}⩾0,\;\sum_{j=0}^{t-1}p_{ij}=1.$$

This satisfies the following conditions:

(3) vP=v

where *t* is the number of states in the Markov chain.

For a Markov chain *M*, its entropy calculation is expressed by Formula (4), as follows:

(4) $$\begin{array}{ll} \mathrm{MCE}(M) & =-\lim_{n\to \infty }\frac{1}{n}\sum_{i_{0},\cdots ,i_{n-1}=0}^{t-1}\left(p_{i_{0}}p_{i_{0}i_{1}}\cdots p_{i_{n-2}i_{n-1}}\right)\cdot \left(\ln p_{i_{0}}p_{i_{0}i_{1}}\cdots p_{i_{n-2}i_{n-1}}\right)\\ \; & =-\lim_{n\to \infty }\frac{1}{n}\sum_{i_{0},\cdots ,i_{n-1}=0}^{t-1}\left(p_{i_{0}}p_{i_{0}i_{1}}\cdots p_{i_{n-2}i_{n-1}}\right)\cdot \left(\ln p_{i_{0}}+\ln p_{i_{0}i_{1}}+\cdots +\ln p_{i_{n-2}i_{n-1}}\right)\\ \; & =-\lim_{n\to \infty }\frac{1}{n}\left(\sum_{i_{0}=0}^{t-1}p_{i_{0}}\ln p_{i_{0}}+\left(n-1\right)\sum_{i,j=0}^{t-1}p_{i}p_{ij}\ln p_{ij}\right)\\ \; & =-\sum_{i,j=0}^{t-1}p_{i}p_{ij}\ln p_{ij}. \end{array}$$

### 2.3. Gene Regulatory Network Reconstruction

We reconstruct the gene regulatory network based on Markov chain entropy and the maximum entropy principle. The principle of maximum entropy, proposed by E.T. Jaynes in 1957, suggests that when only partial knowledge about a distribution is available, the most reasonable inference is the one with the highest entropy, representing the maximum uncertainty [29].

Let us consider a gene regulatory network G={V,E}, where *V* represents the set of *n* nodes and *E* the set of *m* edges. The transition probability matrix is denoted as $P=\{p_{ij}\}$. In the absence of an edge between two distinct nodes, *i* and *j*, $p_{ij}=0$.

The network is assumed to have a steady state with an invariant distribution $\pi =(\pi_{1},\cdots ,\pi_{n})$, determined by the gene expression profiles of the samples. Then, the Markov chain entropy of a Markov transfer on a gene regulatory network *G* of sample *k* is defined as follows:

(5) $$\mathrm{MCE}\left(G^{k}\right)=-\sum_{\left(i,j\right)\in \bar{E}}\pi_{i}p_{ij}\log\left(\pi_{i}p_{ij}\right)$$

Let $\bar{V}$ denote the set of all nodes and $\bar{E}$ denote the set of all edges, including self-loops. In the network, $\pi_{i}p_{ij}$ represents the probability of transitioning from node *i* to node *j* along the directed edge (i,j) in *E*, reflecting the flow of information or the actual interactions. The MCE quantifies the overall entropy of information flow induced by the Markov chain on the network, serving as a measure of network heterogeneity. A higher dynamism in interactions leads to a larger MCE, reflecting greater complexity in information flow.

The dataset is represented by the set $X=\{x^{\left(1\right)},x^{\left(2\right)},\ldots ,x^{\left(q\right)}\}$, where *q* is the sample size and $x^{\left(k\right)}$ is the column vector of gene expression for sample *k* of length *n*.

The adjacency matrix of the gene regulatory network, denoted as *A*, includes self-loops (diagonal elements set to 1) and has a size of n×n. Let *X* represent the data matrix. Before calculating the MCE for a sample, two assumptions are made as follows: (1) the normalized gene expression $\pi =x/{\left||x|\right|}_{L_{1}}$ serves as the invariant distribution of the Markov transition matrix *P* over the network *A*; (2) among all possible transition matrices *P* consistent with π and the topology *A*, the one with the highest entropy is selected.

In complex systems like gene regulatory networks, where precise information is often lacking, the maximum entropy principle enables the construction of an objective probabilistic model under known constraints, capturing the network’s overall structure and behavior from limited data without additional assumptions. Given that π is known, as is the node entropy, the aforementioned equation can be expressed as a standard convex optimization problem with edge entropy as follows:

(6) $$\begin{array}{ll} \mathrm{MCE}\left(G^{k}\right)=\max_{p_{ij}\geq 0}\;- & \sum_{\left(i,j\right)\in \bar{E}}\pi_{i}p_{ij}\log\left(\pi_{i}p_{ij}\right)\\ \;\;\;\;\;\;\;\;\;\;\;\;\;\;\;\;\mathrm{subject}\;\mathrm{to}\;\; & \sum_{j\in \bar{N}\left(i\right)}p_{ij}=1,\;i=1,\ldots ,n\\ \; & \sum_{i\in \bar{N}\left(j\right)}\pi_{i}p_{ij}=\pi_{j},\;j=1,\ldots ,n \end{array}$$

$P={\left(p_{ij}\right)}_{n\times n}$ has the same entries as *A*, with the variables located only at position 1 in *A*. It is possible to change the variables to their Lagrange multipliers and solve the problem as follows [18]:

(7) $$L\left(p_{ij},\lambda ,\mu \right)=\sum_{\left(i,j\right)\in \bar{E}}\pi_{i}p_{ij}\log\left(p_{ij}\right)-\sum_{i}\lambda_{i}\left(\sum_{j\in \bar{N}\left(i\right)}p_{ij}-1\right)-\sum_{j}\mu_{j}\left(\sum_{i\in \bar{N}\left(j\right)}\pi_{i}p_{ij}-\pi_{j}\right).$$

Setting $\alpha_{i}=\exp\left(\lambda_{i}/\pi_{i}\right)$, $\beta_{j}=\exp\left(\mu_{j}-1\right)$, and $p_{ij}=\alpha_{i}\beta_{j}a_{ij}$, the iterative process, initialized with a suitable starting point $\beta^{\left(0\right)}$, continues until $\left(\alpha^{\left(n\right)},\beta^{\left(n\right)}\right)$ converges to a fixed point $\left(\alpha^{*},\beta^{*}\right)$.

Therefore, the transition probability matrix *P* of sample *k* is

(8) $$P\left(k\right)=\alpha^{*}\left(k\right)\beta^{*T}\left(k\right)A$$

where P(k) is the transition probability matrix for sample *k*, and $\alpha^{*}\left(k\right)$ and $\beta^{*}\left(k\right)$ are the Lagrange multipliers for sample *k* that converge to a fixed point through the iterative process.

### 2.4. Markov Flow Entropy and Network Methylation Index

Similar to the Markov chain entropy of the sample defined earlier, the Markov Flow Entropy(MFE) for gene *i* of sample *k* is defined as follows:

(9) $$MFE\left(i^{k}\right)=-\sum_{j=1}^{n}\pi_{i}^{k}p_{ij}^{k}\log\left(\pi_{i}^{k}p_{ij}^{k}\right)$$

where $MFE(i^{k})$ is the Markov Flow Entropy for gene *i* in sample *k*. $\pi_{i}^{k}$ denotes the normalized expression of gene *i* and $p_{ij}^{k}$ represents the transfer probability from the neighboring nodes of gene *i* to gene *j* in sample *k*. A larger value of MFE indicates that the gene is more significantly influenced by other genes.

The MFE values for each gene within networks $G_{0}$ and $G_{1}$ of a specific sample are calculated separately, resulting in $MFE_{0}\left(i\right)$ and $MFE_{1}\left(i\right)$ for gene *i* (please refer to Section 3.2 for specific details on $G_{0}$ and $G_{1}$). The network methylation index, ρ(i), for gene *i* in the weighted gene regulatory network is then determined as follows:

(10) $$\rho \left(i\right)=\frac{MFE_{1}\left(i\right)-MFE_{0}\left(i\right)}{MFE_{0}\left(i\right)}$$

If ρ(i) is less than zero, the effect of the methylation of other genes on gene g is inhibitory; if ρ(i) is greater than zero, the effect of the methylation of other genes on gene *g* is facilitatory; and the greater the value of |ρ(i)|, the more gene *i* is affected by the methylation of other genes.

### 2.5. Weighted Pagerank Algorithm

Weighted PageRank is an algorithmic approach to the ranking and importance assessment of network nodes, typically employed in the context of directed weighted graphs [30]. The objective of this approach is to ascertain the significance of nodes (genes) in accordance with the configuration of links (edges) within the network, with weights that reflect the strength of the connections. Higher in-degrees and edge weights contribute more to the propagation of the PageRank value to the target node. The modified formula is as follows:

(11) $$\mathrm{PR}\left(v\right)=\frac{1-\alpha }{N}+\alpha \sum_{u\in N(v)}w_{uv}\frac{\mathrm{PR}(u)}{C_{\mathrm{in}}\left(u\right)}$$

where PR(v) is the PageRank value of node *v*; α is the damping factor, typically set to 0.85; *N* is the total number of nodes in the graph; N(v) is the set of neighbors of node *v*; $w_{uv}$ is the weight of the edge from node *u* to node *v*; and $C_{\mathrm{in}}\left(u\right)$ is the in-degree of node *u*, which is the number of edges pointing to node *u*.

### 2.6. Core Genes and Bridge Genes

We carried out weighted PageRank clustering on $G_{0}$ and $G_{1}$ independently. This process allowed us to analyze the importance of genes within each network based on their connectivity and the strength of their interactions. After that, we focused on ternary gene sets and recorded the co-occurrence times of these sets. We then selected the top 100 gene triplets with the highest co-occurrence frequencies. Subsequently, we removed duplicate genes from the results obtained from $G_{0}$ and $G_{1}$ separately, generating two distinct gene lists corresponding to $G_{0}$ and $G_{1}$. The final step involved finding the intersection of the genes in these two lists. The resulting gene set is what we defined as the core gene set.

This core gene set has a unique property as follows: it is not significantly influenced by gene methylation regulation. Moreover, the genes in this set have relatively high significance in both the $G_{0}$ (unmethylated) and $G_{1}$ (methylated) networks, suggesting that they play crucial and stable roles in the pathogenesis of the disease, regardless of the methylation state of the genes. These core genes and their first-order neighbors form the core network, at which point bridge nodes (connecting different core genes) surface. Thus, core genes and bridge genes are defined as follows:

- Core genes: Genes with high weighted pagerank scores and consistently appearing in the same clusters in the $G_{0}$ and $G_{1}$ networks of most samples, showing stable expression patterns and playing an important role in disease onset and progression.
- Bridge genes: Genes connecting different core genes, playing a key bridging role in the network and facilitating information transfer between different functional modules.

## 3. Results

### 3.1. Reconstructed Weighted Gene Regulatory Networks Show Improved Clustering Properties

We reconstruct the gene regulatory network based on the Markov chain entropy and maximum entropy principle. In light of the aforementioned findings, we put forth a methodology for constructing weighted gene regulatory networks. This methodology, which is grounded in maximum entropy theory and Markov chain entropy, necessitates only two inputs as follows: the topology of the gene regulatory network and the expression data of the samples.

**Figure 2:**
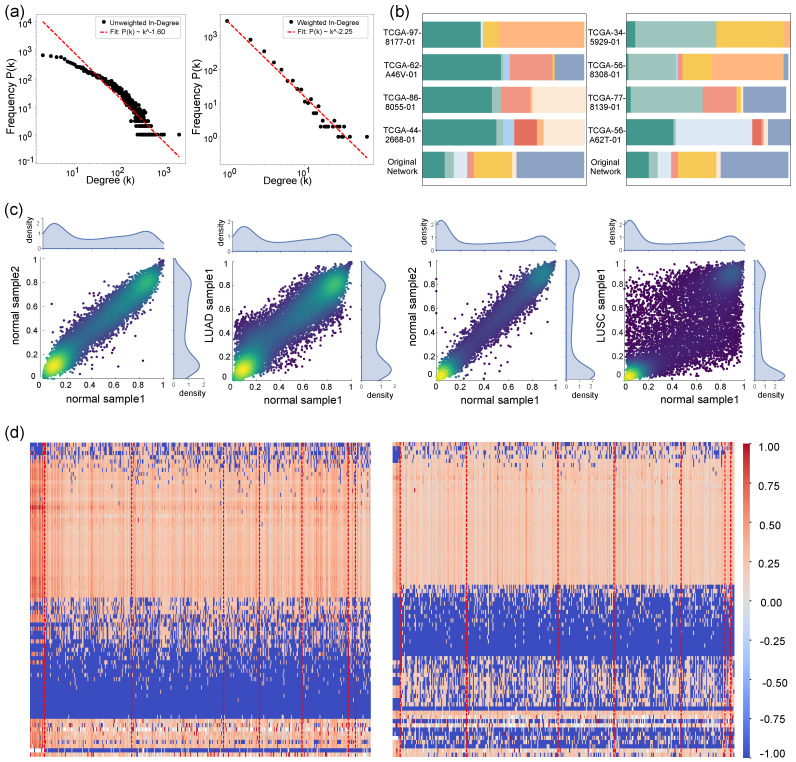
(**a**): Degree distribution of gene regulatory networks after and before gene regulatory network reconfiguration. (**b**): Plot of clustering effects of gene regulatory network driver genes before and after reconfiguration. The original network is the clustering effect before reconfiguration, the other four are the clustering effects of driver genes after gene the regulatory network reconfiguration of the four randomly selected samples from LUAD and LUSC. Different colors represent different cluster categories. (**c**): Distribution of methylation beta values for normal−normal and normal−tumor LUAD and LUSC genes. The two figures on the left show the distribution of LUAD patients and normal samples, while the two figrues on the right show the distribution of LUSC patients and normal samples. (**d**): Heatmap of network methylation index distribution for all samples of LUAD and LUSC, with the area divided by the red dotted line representing the AJCC stage of the disease from left to right as follows: Normal, Stage IA, Stage IB, Stage IIA, Stage IIB, Stage IIIA, Stage IIIB, Stage IV. The colors represent the magnitude of the network methylation index.

A comparison is made between the gene regulatory network and the reconstructed weighted gene regulatory network (for details on calculating the transition probability matrix, see Section 2.3). Prior to reconstruction, the in-degree and out-degree of nodes in the gene regulatory network are both integers and adhere to a power-law distribution. Furthermore, since the network was constructed to satisfy a Markov process, the transfer probabilities of transferring gene *i* to other genes sum to one, whereas the in-degree is a continuous value. Consequently, the interval division is conducted initially in the in-degree statistics, which reveal that the in-degree also adheres to the power-law distribution, as illustrated in Figure 2a.

The reconstructed weighted gene regulatory network exhibits good performance compared to the original network from BioGRID. Separately, before and after reconstruction, the gene regulatory networks were scored using the PageRank algorithm, and the nodes were classified into 10 clusters using Kmeans clustering. The choice of 10 clusters was guided by mathematical and biological considerations. We calculated the average entropy for different numbers of clusters (from 5 to 15) and found them to be positively correlated. However, beyond 10 clusters, the rate of increase in entropy slowed. Grouping genes into 10 clusters ensures that pathogenic genes are neither concentrated in a few clusters nor dispersed excessively, capturing a balance of gene interaction complexity and interpretability.

We viewed the distribution of COSMIC driver genes in the gene regulatory network before and after reconstruction. We found that pathogenic genes were always distributed within three clusters in the gene regulatory network after the reconstruction; meanwhile, the distribution of the gene regulatory network before the reconstruction was very fragmented, as shown in Figure 2b.

The enhanced aggregation of disease-causing genes in reconstituted gene regulatory networks usually reflects their functional relevance and modularity features in disease onset and progression. This phenomenon may indicate that disease-causing genes are collectively involved in specific biological processes or signaling pathways whose regulatory roles or interactions are significantly enhanced in disease states. In addition, such clustering may be associated with the presence of disease driver modules, highlighting the critical role of these modules in disease. This demonstrates the effectiveness of our network construction algorithm and provides new ideas for a wide range of research applications.

### 3.2. Discovering Gene Pathogenicity Patterns via Network Methylation Indices

High levels of DNA methylation, particularly in promoter regions, are widely associated with transcriptional repression. Methylation in these regions hinders the binding of RNA polymerase and transcription factors, leading to gene silencing—a mechanism relevant to processes such as development, cell differentiation, and tumorigenesis. In this study, genes with beta values greater than or equal to 0.6 were classified as fully methylated, less than or equal to 0.2 as unmethylated, and between 0.2 and 0.6 as partially methylated. The initial analysis of methylation data revealed that most samples displayed bimodal distribution patterns, peaking at 0.1 and 0.9, with a subset of genes exhibiting more variable distributions, as shown in Figure 2c.

In accordance with the Law of Centrality, the methylation of DNA exerts a direct influence on gene transcription. If we assume that genes with high methylation levels all repress the transcription and expression of genes, then, we can consider the methylation acting on genes to be a transient effect. In contrast, the subsequent decrease in the expression of genes affected by methylation is a continuous process. In light of the above, we put forth the following two premises:

1. At the moment of $T_{0}$, the weighted gene regulatory network constructed based on the transition matrix arrived at by the expression values of all genes is $P_{0}$, which is regarded as the weighted gene regulatory network $G_{0}$, is unaffected by methylation;
2. Based on the previous assumption that the $T_{1}$ moment is affected by methylation, we subtract the genes with high methylation levels from the network, and we obtain the transition matrix following the methylation effect as $P_{1}$ and the weighted gene regulatory network of methylation effect $G_{1}$.

In accordance with these two premises, we constructed two distinct weighted gene regulatory networks as follows: $G_{0}$, which is unmethylated, and $G_{1}$, which is methylated. Each network was constructed for a specific sample. Given that the network was constructed to satisfy a Markov process, the transfer probabilities of transferring gene *i* to other genes must sum to one. In this context, $p_{ij}$ represents the out-degree, or the probability of gene transfer from other genes to gene *i*. The in-degree, or the probability of gene transfer from gene *i* to other genes, is not yet determined. For gene *i*, the out-degree sum is 1, while the in-degree sum is uncertain. Consequently, the degree to which gene *i* is influenced by the regulation of other genes is quantified by the in-degree. The MFE and network methylation index ρ(i) for gene *i* is defined in Section 2.4. The network methylation index was calculated to compare the role of gene methylation in the network by contrasting the MFE values of genes in $G_{0}$ and $G_{1}$.

The Tier 1 phase-specific causal genes associated with LUAD, as identified in the COSMIC database, were analyzed, and the results are presented in Figure 2c. The red dashed lines in the figure indicate progression through distinct disease stages, arranged from left to right as follows: “Normal”, “Stage IA”, “Stage IB”, “Stage IIA”, “Stage IIB”, “Stage IIIA”, “Stage IIIB”, and “Stage IV”. In LUAD, the majority of normal samples exhibited high methylation levels in genes such as *ERBB2*, *ROS1*, *SDC4*, *PIK3CB*, and *MYCL*, whereas disease samples predominantly demonstrated low methylation levels for these genes. Similarly, genes including *DROSHA*, *SIRPA*, *HIF1A*, and *MAP2K1* showed a similar pattern, with high methylation levels in most normal samples and low methylation levels in disease samples, though the proportion of disease samples exhibiting low methylation remained relatively small.The methylation profile of *FGFR2* revealed a distinct pattern, with a high proportion of normal samples exhibiting elevated methylation levels, while disease samples showed a comparatively low proportion of high methylation levels. Moreover, *FGFR2* and *KDR* were notable for their absence of a high methylation index in normal samples, yet both demonstrated a significant proportion of highly methylated cases in disease samples. This distinct methylation shift underscores their potential role in disease progression.

Therefore, it can be demonstrated that those genes, which are pathogenic due to methylation, can be divided into two categories as follows:

- Genes that cause cancer due to methylation: *FGFR2* and *KDR*.
- Genes that restrain cancer due to methylation: *ERBB2*, ROS1, *SDC4*, PIK3CB, *MYCL*, DROSHA, *SIRPA*, HIF1A, and *MAP2K1*.

Among these genes, the overexpression of *FGFR1* is driven by both genetic and epigenetic mechanisms [31]. *KDR* promotes tumor angiogenesis, while the hypermethylation of the Keap1 promoter reduces *KEAP1* expression, contributing to tumor progression [32]. Methylation changes in *ERBB2* and *PIK3CB* affect gene expression and play roles in LUAD development. The *EML4-ALK* fusion gene is a key oncogenic driver in NSCLC [33,34]. Additionally, the genetic or epigenetic loss of *NKX2-1* is important in LUAD development. In the A549 cell line, the upregulation of *SLC34A2* inhibits cell viability and invasion, suggesting its potential as a therapeutic target [35]. These findings underscore the importance of both genetic and epigenetic alterations in NSCLC.

Similarly, as shown in the left figure of Figure 2d, genes that become pathogenic genes due to gene methylation in LUSC can also be divided into two categories as follows:

- Genes that cause cancer due to methylation: *KEAP1*, *EML4*, *SIRPA*, *FGFR2*, HIP1, *NOTCH1*, *NKX2-1*, and *SLC34A2*.
- Genes that restrain cancer due to methylation: *LRIG3*, *KRAS*, *HIF1A*, *SOX2*, *BRAF*, *NFE2L2*, *EGFR*, and *DROSHA*.

In these genes, the hypermethylation of the Keap1 promoter leads to decreased *KEAP1* mRNA and protein expression, contributing to tumor progression [36]. The *EML4-ALK* fusion gene plays a key oncogenic role in NSCLC, while the genetic or epigenetic inactivation of *NKX2-1* may impact the development and abnormal differentiation of NSCLC [37]. The methylation of genes such as *RUNX3*, *GSTP1*, *SHOX2*, *DKK3*, and *MLH1* is associated with tumor progression and malignancy, with RUNX3 showing high promoter methylation in NSCLC tissues [38,39,40]. Additionally, the methylation status of *SPARC* differs between LUAD and LUSC, while the abnormal methylation of *SOX1* promoters is observed in the serum of NSCLC patients [41]. *EGFR* expression is inversely correlated with methylation levels and poorly differentiated tumors, showing low methylation, whereas well-differentiated tumors exhibit higher methylation [42]. Moreover, the methylation of the MGMT promoter is linked to TNM staging, lymph node metastasis, and tumor differentiation in NSCLC. These epigenetic alterations highlight the significant role of DNA methylation in NSCLC tumorigenesis and progression.

### 3.3. Methylation Networks Show Potential Tumor Genes

After analyzing the role of methylation in pathogenic genes, we found genes with severe network methylation index absence in pathogenic genomes and constructed methylation core networks with these genes and their first-order neighbors. By analyzing the regulatory relationships and bridge nodes of each methylation core network, we found several potential genes that affect LUAD and LUSC through methylation, see Table 2.

In the methylation core networks of LUAD, the *EML4* and *DROSHA* genes are identified as Tier 1 driver genes in COSMIC, while the *RFWD3* and *N4BP2* genes are both Tier 2 driver genes in COSMIC. Based on that, the first-order neighbors of these four genes are identified, and the network composed of these genes is defined as the LUAD core subnetwork. It can be demonstrated that the network comprising the three genes *DROSHA*, *EML4*, and *RFWD3* and their first-order neighbors is connected. Concurrently, *KRCC1* connects the three disease-causing genes *DROSHA*, *EML4*, and *RFWD3*. *EVA1C*, *TRAJ56*, *IL18*, *AURKA*, and *G3BP1* were found to concurrently regulate the two disease-causing genes *EML4* and *RFWD3*. Similarly, *UBA5*, *CAD*, *CCDC102B*, *GLG1*, *PPP2R2D*, and *TSPAN13* were identified as regulators of the two pathogenic genes, *DROSHA* and *RFWD3*. *ADH6* was found to be regulated by *EML4*, which in turn regulated *DROSHA*.

**Figure 3:**
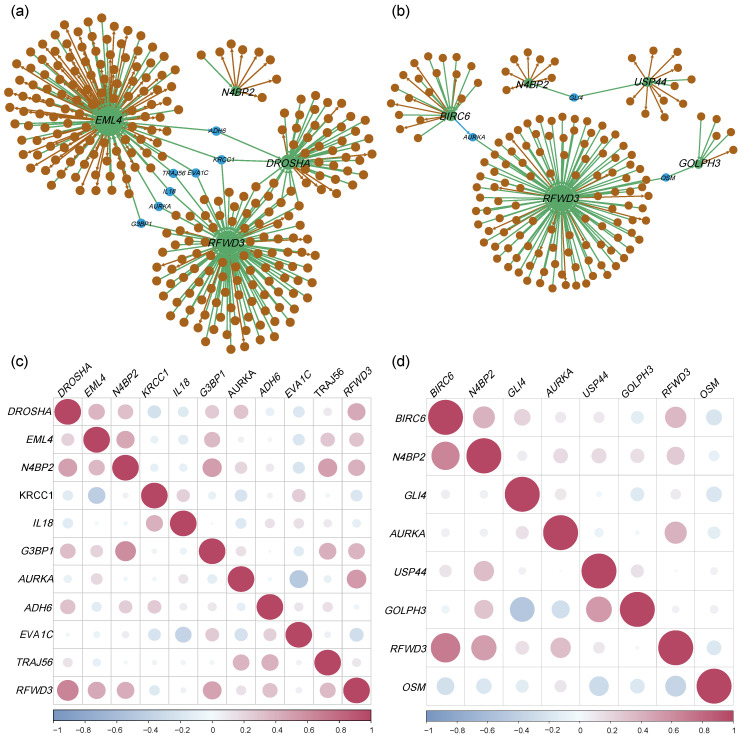
(**a**,**b**): Methylation network of LUAD (**a**) and LUSC (**b**). Green nodes represent the severely methylated genes in most of the samples and blue nodes are bridge nodes. The other nodes are normal genes. The arrows are colored the same as their target genes. (**c**,**d**): Heatmap of correlation between normal and tumor samples for both LUAD (**c**) and LUSC (**d**) cancers. Separated by diagonal lines, the lower half is for normal samples and the upper half is for tumor samples.

As illustrated in Figure 3a, several genes exhibited notable shifts in correlation patterns in the LUAD tumor samples, highlighting potential alterations in their functional roles. *DROSHA* transitioned from a negative to a positive correlation with *AURKA*, while intensifying its negative correlation with *EVA1C*. *EML4* and *N4BP2* displayed similar shifts, transitioning from negative to positive correlations with *TRAJ56*, with *EML4* also showing a reduced negative correlation with *KRCC1*. *KRCC1* exhibited varying correlations with TRAJ56 and *EVA1C*, spanning from negative to positive. *IL18* and *G3BP1* showed both negative and positive correlations with *EVA1C* and *AURKA*, respectively, while *RFWD3* consistently demonstrated reduced expression and diminished correlation with *TRAJ56*. *ADH6* displayed fluctuating correlations with *EVA1C*, *TRAJ56*, and *RFWD3*, indicating its complex regulatory role.

These findings align with the known roles of these genes in lung cancer. *DROSHA* acts as a context-dependent regulator, functioning as a tumor suppressor in some cases while contributing to tumor progression through miRNA expression changes in others [43]. *EML4*, frequently involved in oncogenic fusion events like *EML4-ALK*, is critical in NSCLC pathogenesis and responsive to targeted therapies [44]. *N4BP2* is implicated in fibrotic processes and angiogenesis, potentially influencing tumor microenvironments [45]. *KRCC1*, often overexpressed in malignancies, is associated with poor prognosis and impacts tumor growth and apoptosis. Similarly, *RFWD3* plays a pivotal role in the DNA damage response, stabilizing p53 and regulating cell cycle checkpoints [46]. These shifts in correlation patterns suggest altered gene interactions and regulatory dynamics in tumor samples, providing potential insights into disease mechanisms.

The five genes in the methylation core networks of LUSC—*BIRC6*, *N4BP2*, *RFWD3*, *GOLPH3*, and *USP44*—have been classified as Tier 2 cancer-causing genes. Based on these genes, their first-order neighbors were identified to construct the LUSC core subnetwork. Notably, two subnetworks can be observed as follows: one formed by *DROSHA*, *EML4*, and *BIRC6*, along with their first-order neighbors; and another composed of *N4BP2* and *USP44*. These subnetworks exhibit connectivity within the larger LUSC network. Key regulatory interactions include *AURKA*, regulated by *BIRC6* and acting as a regulator of *RFWD3*; OSM, which regulates both *RFWD3* and *GOLPH3*; and *GLI4*, which simultaneously regulates *N4BP2* and *USP44*. The analysis of Figure 3d reveals that the expression correlation between *BIRC6* and *GLI4* increases in tumor samples. Additionally, the negative correlation between *GOLPH3* and *GLI4*, as well as *AURKA*, becomes significantly weaker in tumor samples. Furthermore, the positive correlation between *GOLPH3* and *USP44* in tumor samples also decreases substantially. Similarly, the negative correlation between *OSM* and *USP44* in tumor samples is notably reduced.

Functionally, these genes play critical roles in lung cancer progression. The overexpression of *BIRC6*, a member of the inhibitor of apoptosis (IAP) family, contributes to chemoresistance and poor prognosis by inhibiting apoptosis, making it a promising therapeutic target [47]. *GOLPH3*, frequently amplified in NSCLC, functions as an oncogene, facilitating Golgi secretory trafficking and driving tumorigenesis and cellular transformation [48]. In contrast, *USP44* acts as a tumor suppressor, with its deficiency linked to spontaneous lung tumor development in mouse models, underscoring its protective role against tumorigenesis [49]. These findings highlight the complex interplay between oncogenic drivers and tumor suppressors within the LUSC core subnetwork, offering insights into potential therapeutic interventions.

### 3.4. Identification of a Stable Core Pathogenic Gene Set in LUAD and LUSC

A weighted PageRank clustering analysis was conducted on all constructed networks to identify groups of genes that appeared in the same cluster at the same time; see Section 2.5 for the specific steps. We performed weighted PageRank clustering on $G_{0}$ and $G_{1}$, respectively, then extracted the co-occurrence times of the ternary gene sets and took the gene triplets with the top 100 co-occurrence times. After deduplication, we obtained two gene lists for $G_{0}$ and $G_{1}$, respectively. Finally, we took the intersection of the genes in the two lists to obtain a core pathogenic gene set. The gene set extracted through the above steps is not affected by gene methylation regulation, and it is relatively high in both $G_{0}$ and $G_{1}$. We call this the stable core pathogenic gene set. We took the stable core pathogenic gene set as the key, merged their first-order neighbors to form a network, and focused on analyzing the core nodes and bridge nodes in the network. The table shows the core genes and bridge genes of LUAD and LUSC. For the definitions of core genes and bridge genes, refer to Section 2.6. All core and bridge genes of LUAD and LUSC are listed in Table 3 and Table 4.

**Figure 4:**
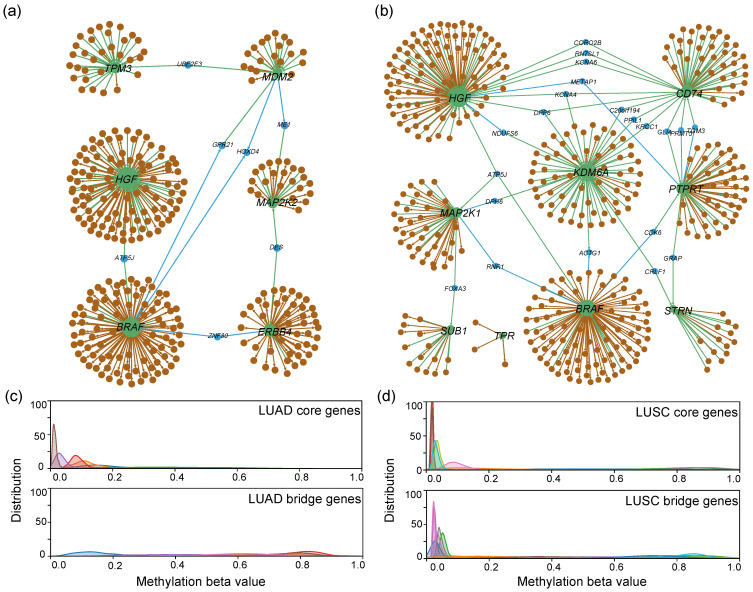
(**a**,**b**): Subnetworks consisting of a stable core set of pathogenic genes for LUAD (**a**) and LUSC (**b**), where green nodes indicate core genes, blue nodes indicate bridge genes, and the size of the node represents the degree of the node. The other nodes are normal genes. The arrows are colored the same as their target genes. (**c**,**d**): Distribution of methylation beta values for core and bridge genes for LUAD (**c**) and LUSC (**d**). Different colors represent different genes.

As can be seen from Figure 4b, the methylation beta values of the stable core genes we found are mainly distributed below 0.2, and they are indeed not affected by methylation. However, the bridge genes in the LUAD network have no beta values in most samples, so the subsequent analysis focuses on the impact of the core genes and bridge genes of LUSC on the disease.

**Figure 5:**
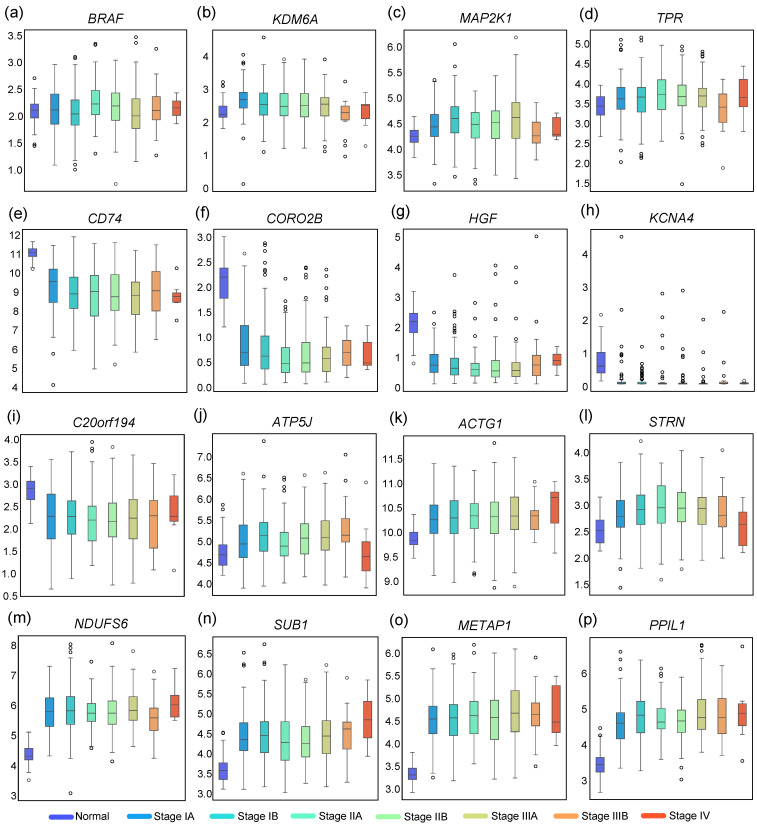
(**a**–**p**): Boxplots of the expression of some LUSC core and bridge genes by AJCC stage, the horizontal coordinates are the different stages in the following order: ‘Normal’, ‘Stage IA’, ‘Stage IB’, ‘Stage IIA’, ‘Stage IIB’, ‘Stage IIIA’, ‘Stage IIIB’, and ‘Stage IV ’, and the expression was FPKM. The meaning of the colors is shown in the legend at the bottom of the picture.

As shown in Figure 4a,b, the number of core and bridge genes in LUSC is much larger than that of LUAD. The next step focuses on analysing the network of LUSC. The core pathogenic sub-network of LUSC is shown in Figure 4b. The expression of certain genes shows significant changes during the progression from normal tissue to cancer, classified by stages. From Figure 5a–d, it is evident that the expression levels of genes *BRAF*, *KDM6A*, *MAP2K1*, and *TPR* show minimal variation from Normal to Stage IV. However, these genes play crucial roles in NSCLC. *BRAF* mutations drive oncogenesis through the MAPK pathway and are established therapeutic targets in NSCLC [50]. Silencing *KDM6A* suppresses tumorigenic phenotypes in NSCLC cell lines [51]. *MAP2K1* variations influence signal transduction, cell proliferation, and metastasis, impacting lung cancer progression and treatment. TPR-related mutations, including TPR-ALK fusions, may initiate lung cancer and serve as potential biomarkers for immunotherapy.

By observing Figure 5e–i, we can deduce that *CD74*, *HGF*, *MAP2K1*, *CORO2B*, *KCNA4*, *DPP6*, and *C20orf194* exhibit a marked decrease from normal tissue to Stage IA. Conversely, genes such as *MAP2K1*, *SUB1*, *CDK6*, *METAP1*, *GLI4*, *PRMT5*, *DPH6*, *ACTG1*, *PPIL1*, and *NDUFS6* show a significant increase in expression during the same transition. In later stages, genes like *STRN* and *ATP5J* demonstrate reduced expression in Stage IV LUSC, while *SUB1*, *DPH6*, and *ACTG1* are upregulated at Stage IV, suggesting their potential as biomarkers for disease progression. *CD74*, *HGF*, and *SUB1* are frequently overexpressed in NSCLC, promoting tumor proliferation and survival. The knockdown of *CD74* or *SUB1* suppresses tumor growth, and *HGF* has shown potential as a therapeutic target due to its role in fibrosis reversal and lung repair. Interestingly, gene *CD74* was overexpressed in NSCLC in most of the studies; however, with the results of our analysis of the TCGA-LUSC dataset, the expression of *CD74* was significantly decreased in the tumor samples.

## 4. Discussion

In this study, we developed a downstream analysis method based on known gene regulatory networks from the BioGRID database, integrating RNA-seq and methylation data to assign weights to regulatory relationships and reconstruct networks. Unlike de novo network inference methods, our approach leverages existing biological knowledge, providing a more accurate representation of gene regulation in the context of lung cancer. Compared to the original gene regulatory network, our method demonstrates enhanced performance in clustering pathogenic genes. Furthermore, by comparing $G_{0}$ (non-methylated network) and $G_{1}$ (methylated network), we identify functional modules and analyze the methylation effects. For instance, we identified stable core pathogenic genes (e.g., *BRAF*, *KDM6A*) and methylation-driven oncogenes (e.g., *FGFR2*, *KDR*), which were not effectively captured in the original network, while our method focuses on the downstream analysis of known networks, future work will include benchmarking against existing inference methods to further validate its advantages. This approach offers a robust framework for uncovering key regulatory mechanisms in complex diseases.

The integration of gene expression and DNA methylation data in this study represents a significant advancement in understanding the complex mechanisms underlying lung cancer. By employing a novel weighted gene regulatory network reconstruction method based on maximum entropy principles and Markov chain entropy, we were able to build biologically informed networks that exhibit power-law distributions and enhance the clustering of pathogenic genes. This reflects the critical modularity and functional relevance of disease-associated genes in cancer progression.

We applied this reconstructed gene regulatory network method to LUAD and LUSC datasets, constructing an original network $G_{0}$ and a methylation-specific network $G_{1}$ for each sample. By defining a network methylation index, we analyzed the impact of DNA methylation on known pathogenic genes during disease progression, classifying these methylation-associated pathogenic genes into two categories as follows: methylation-driven oncogenes and methylation-driven tumor suppressor genes. This approach provides a more nuanced understanding of how DNA methylation contributes to cancer progression, revealing potential targets for epigenetic therapies. By distinguishing between the oncogenic and tumor-suppressive roles of methylation, this method offers valuable insights into the dual nature of epigenetic regulation in cancer and lays the foundation for more precise diagnostic and therapeutic strategies.

The analysis of the network methylation index for LUAD and LUSC highlights the intricate regulatory interplay between key driver genes and their first-order neighbors. The observed shifts in correlation patterns and the identification of regulatory nodes provide valuable insights into the altered gene interactions and functional dynamics in tumor samples. These findings emphasize the dual roles of certain genes, functioning as either oncogenic drivers or tumor suppressors, depending on the context. Furthermore, the identification of central regulators, such as *AURKA*, *OSM*, and *GLI4*, underscores their potential as therapeutic targets.

By focusing on the core and bridge genes in the network of the stable core pathogenic gene set, this work advances our understanding of the molecular pathways underlying lung cancer progression, paving the way for the development of more personalized and effective targeted therapies. Our method can not only uncover disease-causing genes with abnormal expression in tumor samples such as *CD74*, *HGF*, but it can also identify genes with stable expression yet crucial roles in LUSC, such as BRAF, KDM6A, MAP2K1, and *TPR*. In the analysis of both the methylation network and the network of stable core pathogenic genes, *KRCC1* consistently emerged as a bridge gene. *KRCC1*, often overexpressed in various malignancies, is associated with poor prognosis and plays a role in promoting tumor growth and inhibiting apoptosis. These findings suggest that *KRCC1* may serve as a novel biomarker for NSCLC. Meanwhile, the *MAP2K1* and *MAP2K2* genes regulate cell proliferation and survival through the MAPK signaling pathway, and their abnormal activation drives tumor growth, metastasis, and drug resistance in lung cancer, making them critical therapeutic targets.

**Figure 6:**
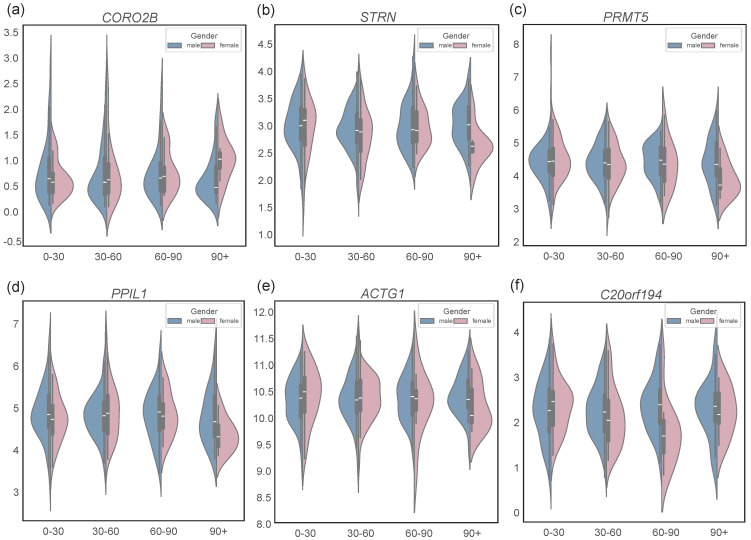
(**a**–**f**) Violin plots of LUSC partial core and bridge genes by sex in response to smoking. Horizontal coordinates are pack-years of smoking, 0–30, 30–60, 60–90, 90+, respectively. The genes in the figure show different forms of expression in smoking amount and gender.

The application of this methodology to LUSC datasets further demonstrated the impact of gender and smoking status on gene expression patterns, as shown in Figure 6. With increasing cumulative smoking exposure, the expression of *BRAF* remains stable in male patients. However, in female patients, *BRAF* expression is comparable to males when smoking exposure is below 60 pack-years but declines significantly beyond this threshold, with the most pronounced differences observed at 90 pack-years. In contrast, *CD74* expression in male patients is unaffected by smoking exposure but increases in females with over 60 pack-years, correlating with higher exposure levels. Notably, *CORO2B* expression shows a sharp upregulation in female patients with over 90 pack-years, while the expression of *KDM6A* is not influenced by smoking exposure, and it is consistently higher in female patients compared to males. Genes such as *STRN*, *PRMT5*, *PPIL1*, and *ACTG1* maintain stable expression levels in male patients regardless of smoking exposure, but *STRN* exhibits a sharp downregulation in females with more than 90 pack-years of exposure. Similarly, *C20orf194* expression remains unaffected in male patients but shows a significant reduction in females with 60–90 pack-years of exposure compared to their male counterparts. *CORO2B* is associated with smoking-induced malignant transformation, while *C20orf194* shows differential expression in female patients under high smoking exposure, indicating its potential role in gender-specific tumorigenesis.

Despite these advancements, there are limitations to this study. The computational time required for network reconstruction on a per-sample basis can be substantial, making it challenging to handle large datasets efficiently. Furthermore, the current approach to multi-omics integration is limited to its ability to incorporate a broader range of omics data, such as proteomics and metabolomics, which could provide a more comprehensive understanding of cancer mechanisms. Additionally, the choice of clustering parameters lacks rigorous optimization, and the method is sensitive to initial conditions, potentially affecting the robustness of the results. The complexity of the reconstructed networks and the lack of experimental validation also pose challenges in interpreting the biological relevance of the findings. Finally, incomplete clinical data and the method’s focus on lung cancer raise questions about its generalizability to other cancer types or diseases.

To address these limitations, future studies should focus on optimizing computational efficiency to support larger datasets and exploring methods to integrate additional omics data, such as proteomics and metabolomics. Advanced clustering techniques and validation metrics should be employed to improve the robustness of the results. The experimental validation of the identified driver genes and methylation effects, along with the development of tools for better network visualization and interpretation, could enhance the method’s practicality and interpretability. Extending the approach to other cancer types and diseases would further assess its generalizability and potential for broader applications in precision medicine.

## 5. Conclusions

This study introduces an innovative approach to weighted gene regulatory network reconstruction, addressing the limitations of single-omics data and enhancing the clustering of pathogenic genes to uncover critical insights into lung cancer progression. Through the integration of gene expression and DNA methylation data, we identified a stable core pathogenic gene set and key bridge genes, offering valuable biomarkers and therapeutic targets for LUAD and LUSC. The incorporation of methylation-driven oncogenes and tumor suppressor genes into network analyses provides a more comprehensive understanding of the dual nature of epigenetic regulation in cancer.

Our findings also reveal the influence of gender and smoking status on the expression of key genes, underscoring the importance of personalized approaches in lung cancer diagnosis and treatment. By highlighting the central regulatory genes and their roles in tumor progression, this work lays the groundwork for the development of targeted therapies and novel diagnostic strategies. Future research should focus on experimental validation and the integration of multi-omics data to further enhance the understanding of lung cancer mechanisms and improve therapeutic outcomes.

## Figures and Tables

**Table 1:** Statistical information for TCGA-LUAD and TCGA-LUSC, including gender, smoking status (pack years smoked), and stage (Ajcc stage). Some of the samples had missing clinical information.

Gender	Male				Female			
LUAD	191				228			
LUSC	274				96			
**Smoking**	**0–30**		**30–60**		**60–90**		**90+**	
LUAD	120		119		22		25	
LUSC	259		153		49		32	
**Stage**	**Normal**	**IA**	**IB**	**IIA**	**IIB**	**IIIA**	**IIIB**	**IV**
LUAD	18	109	115	45	53	58	9	19
LUSC	8	71	98	60	72	47	6	4

**Table 2:** Driver genes with more deletions in the network methylation index.

LUAD		LUSC	
**Gene**	**Number of Absence**	**Gene**	**Number of Absence**
*N4BP2*	437	*USP44*	369
*RFWD3*	435	*GOLPH3*	366
*DROSHA*	228	*BIRC6*	364
*EML4*	182	*N4BP2*	347
		*RFWD3*	319

**Table 3:** Core and bridge genes of LUAD and their functions.

Gene Symbol	Gene Type	Function
*ERBB4*	Core	Cell signaling in tumorigenesis
*BRAF*	Core	Activates MAPK pathway, oncogenesis
*HGF*	Core	Promotes repair, potential therapeutic
*TPM3*	Core	Cytoskeletal dynamics, metastasis
*MDM2*	Core	Negatively regulates p53
*MAP2K2*	Core	Impacts MAPK signaling, resistance
*UBE2E3*	Bridge	Regulates ubiquitin-proteasome pathway
*ME1*	Bridge	Supports metabolic reprogramming
*ATP5J*	Bridge	Mitochondrial energy metabolism
*GPR21*	Bridge	Cell migration signaling
*HOXD4*	Bridge	Cellular differentiation
*ZNF80*	Bridge	Transcriptional regulation
*DES*	Bridge	Cytoskeletal integrity, metastasis

**Table 4:** Core and bridge genes of LUSC and their functions.

Gene Symbol	Gene Type	Function
*BRAF*	Core	Activates MAPK pathway, oncogenesis
*CD74*	Core	Promotes proliferation, migration
*HGF*	Core	Promotes repair, potential therapeutic
*KDM6A*	Core	Epigenetic regulation, poor prognosis
*MAP2K1*	Core	Disrupts signaling, metastasis
*PTPRT*	Core	Negatively regulates proliferation
*STRN*	Core	Fusion genes, tumorigenesis
*SUB1*	Core	Amplified, promotes tumorigenesis
*TPR*	Core	Fusion genes, poor prognosis
*CDK6*	Bridge	Regulates cell cycle progression
*METAP1*	Bridge	Protein maturation
*GLI4*	Bridge	Modulates Hedgehog signaling
*TGM3*	Bridge	Cellular adhesion, metastasis
*PRMT5*	Bridge	Tumor progression, resistance
*RNR1*	Bridge	Regulates ribosomal function
*ATP5J*	Bridge	Mitochondrial energy metabolism
*DPH6*	Bridge	Translation elongation
*FOXA3*	Bridge	Epithelial differentiation
*CRLF1*	Bridge	Cytokine signaling
*ACTG1*	Bridge	Cytoskeletal dynamics
*KCNA6*	Bridge	Ion channel regulation
*CORO2B*	Bridge	Cigarette smoke-induced transformation
*RN7SL1*	Bridge	Modulates RNA stability
*KCNA4*	Bridge	Regulates potassium ion flux
*DPP6*	Bridge	Proteolytic processes
*PPIL1*	Bridge	Protein folding
*KRCC1*	Bridge	DNA repair
*C20orf194*	Bridge	Transcriptional regulation
*NDUFS6*	Bridge	Electron transport
*GRAP*	Bridge	Regulates signaling pathways

## Data Availability

The BioGrid data can be found at https://downloads.thebiogrid.org/BioGRID/Release-Archive/BIOGRID-4.4.226/ (accessed on 10 November 2023). TCGA-LUAD and TCGA-LUSC can be found at The Cancer Genome Atlas (TCGA) Research Network: http://cancergenome.nih.gov (accessed on 23 November 2023).

## Abbreviations

The following abbreviations are used in this manuscript:

MCE	Markov Chain Entropy
MFE	Markov Flow Entropy
